# Identification of autophagy-related biomarkers in patients with pulmonary arterial hypertension based on bioinformatics analysis

**DOI:** 10.1515/med-2022-0497

**Published:** 2022-07-06

**Authors:** Zhisong Yang, Li Zhou, Haiyan Ge, Weimin Shen, Lin Shan

**Affiliations:** Department of Emergency, Daqing Longnan Hospital, Daqing, Heilongjiang 163453, China; Department of Respiratory Medicine, Shanghai Huadong Hospital, Shanghai 200040, China

**Keywords:** cardiopulmonary disease, congenital heart disease, hub genes, bioinformatics analyses, autophagy

## Abstract

Autophagy participates in the regulation of pulmonary arterial hypertension (PAH). However, the role of autophagy-related genes (ARGs) in the pathogenesis of the PAH is still unclear. This study aimed to identify the ARGs in PAH via bioinformatics analysis. A microarray dataset (GSE113439) was downloaded from the Gene Expression Omnibus database to identify differentially expressed ARGs (DEARGs). Protein–protein interactions network, gene ontology, and Kyoto Encyclopedia of Genes and Genomes enrichment analyses were performed to screen hub genes and the underlying molecular mechanisms of PAH. Finally, the mRNA expression of the hub genes was validated using the GSE53408 dataset. Twenty-six DEARGs were identified, all of which were upregulated. Enrichment analyses revealed that these DEARGs were mainly enriched in the nucleotide-binding oligomerization domain (NOD)-like receptor signaling pathway, PI3K-Akt signaling pathway, response to hypoxia, response to nutrient levels, and autophagy. Among these hub genes, the mRNA expression levels of HSP90AA1, HIF1A, MET, IGF1, LRRK2, CLTC, DNM1L, MDM2, RICTOR, and ROCK2 were significantly upregulated in PAH patients than in healthy individuals. Ten hub DEARGs were identified and may participate in the pathogenesis of the PAH via the regulation of autophagy. The present study may provide novel therapeutic targets for PAH prevention and treatment and expand our understanding of PAH.

## Graphical abstract

**Figure:**
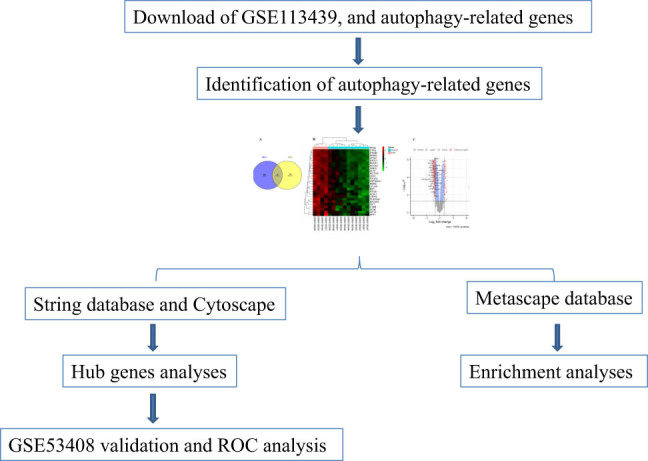

## 1 Introduction

Pulmonary arterial hypertension (PAH) is a multifactorial cardiopulmonary disease, in which vascular thickening is one of the main factors that cause right ventricle failure and cardiac dysfunctions [1]. The annual prevalence of PAH is 15–50 cases per million people, and its incidence is 5–10 cases per million people [2]. Based on clinical classification criteria, PAH can be divided into toxin- and drug-evoked PAH, heritable PAH, idiopathic PAH, PAH with congenital heart disease (CHD), PAH with portal hypertension, PAH with the connective disease, and so on [3]. Patients with PAH and cardiomyopathy usually have a poor prognosis, compared to patients with simple cardiomyopathy, the incidence of atrial fibrillation was higher [4,5]. The pathogenesis of PAH is complex and there is no curative treatment therapy at present. In recent years, advances in effective drug therapy and the use of combination therapy have significantly improved the prognosis of patients with PAH [6,7]. Although previous reports have demonstrated that many biomarkers and related pathways were implicated in the pathological process of PAH, the precise molecular mechanism is still not fully understood [8,9]. Therefore, the investigation of novel biomarkers for PAH is not only important to better understand the pathobiology of PAH, but is also an important way to establish promising new treatment strategies [10].

Autophagy is an important and complex homeostasis process implicated in survival mechanisms and cellular homeostasis in eukaryotes [11]. The impaired level of autophagy participates in the regulation of various diseases, including neurodegenerative diseases, cancer, metabolic diseases, and chronic respiratory disease [12]. Previous reports have demonstrated that lung cancer, lung injury, PAH, idiopathic pulmonary diseases, and other pulmonary diseases are associated with abnormal autophagy [13]. For example, PDIA6 was reported to modulate cisplatin-induced autophagy and apoptosis in non-small cell lung cancer by inhibiting the JNK/c-Jun signaling pathway [14]. Ghrelin was revealed to restrain chronic obstructive pulmonary disease-associated autophagy and inflammation [15]. Moreover, it has been demonstrated that nuclear receptor binding SET domain 2 (NSD2) participates in the progression of monocrotaline-evoked PAH via the regulation of autophagy and trehalose metabolism [16]. Adenosine monophosphate-activated protein kinase (AMPK) activation suppresses the development of PAH via inhibiting vascular remodeling and autophagy [17]. Therefore, identifying the autophagy-related genes may provide us with potential therapeutic targets for PAH. To date, there are still few reports about PAH and autophagy, as most of them only focused on autophagy-related targets *in vivo* or *in vitro*.

The present study aimed to investigate the differentially expressed autophagy-related genes (DEARGs) of PAH from the GSE113439 dataset. Protein–protein interaction (PPI) network was constructed by using Cytoscape software to identify hub genes. Kyoto Encyclopedia of Genes and Genomes (KEGG) enrichment analyses were carried out to investigate the molecular mechanisms of PAH.

## 2 Materials and methods

### 2.1 Data resources

The GSE113439 and GSE53408 datasets were downloaded from the Gene Expression Omnibus database. GSE113439 contained 11 normal controls and four patients with PAH and CHD. GSE53408 contained 11 healthy individuals and 12 severe pulmonary arterial hypertension patients.

### 2.2 Identification of DEARGs

The limma package in R software was used to screen the DEGs between normal samples and PAH samples. |log2FC| ≥1 and *P*-value <0.05 was defined as criteria for DEGs identification [18]. The autophagy-related genes (ARGs) were collected from the Human Autophagy-dedicated Database (http://hamdb.scbdd.com/). Then, we used a Venn diagram package to identify DEARGs. The “ggplot2” and “heatmap” packages of R software were used to draw the volcano plot and heatmap.

### 2.3 KEGG and gene ontology (GO)-biological process (BP) enrichment analyses

KEGG pathway and GO-BP enrichment analyses were carried out using Metascape (http://metascape.org/). The result with *P* < 0.05 was considered statistically significant. The visualization of the enriched pathways was carried out using the clusterProfiler (4.2.1) package in R software.

### 2.4 PPI network construction and hub genes identification

STRING database and Cytoscape software were used to construct and visualize the PPI network of DEARGs. Cytohubba plugin in the Cytoscape was used to identify the hub genes based on the degree algorithm [19].

### 2.5 Statistical analysis

Gene expression values between control and PAH samples were compared using Student’s *t*-tests. *P* < 0.05 was considered statistically significant. The receiver operating characteristic (ROC) was carried out to access the diagnostic value of potential targets, with the area under the curve (AUC) indicating specificity and sensitivity.


**Ethics approval and informed consent**: The present study was carried out based on public databases, so approval was not required for this study by the ethics committee and there are no ethical issues or other conflicts of interest.

## 3 Results

### 3.1 Identification of DEGs and DEARGs

**Figure 1:**
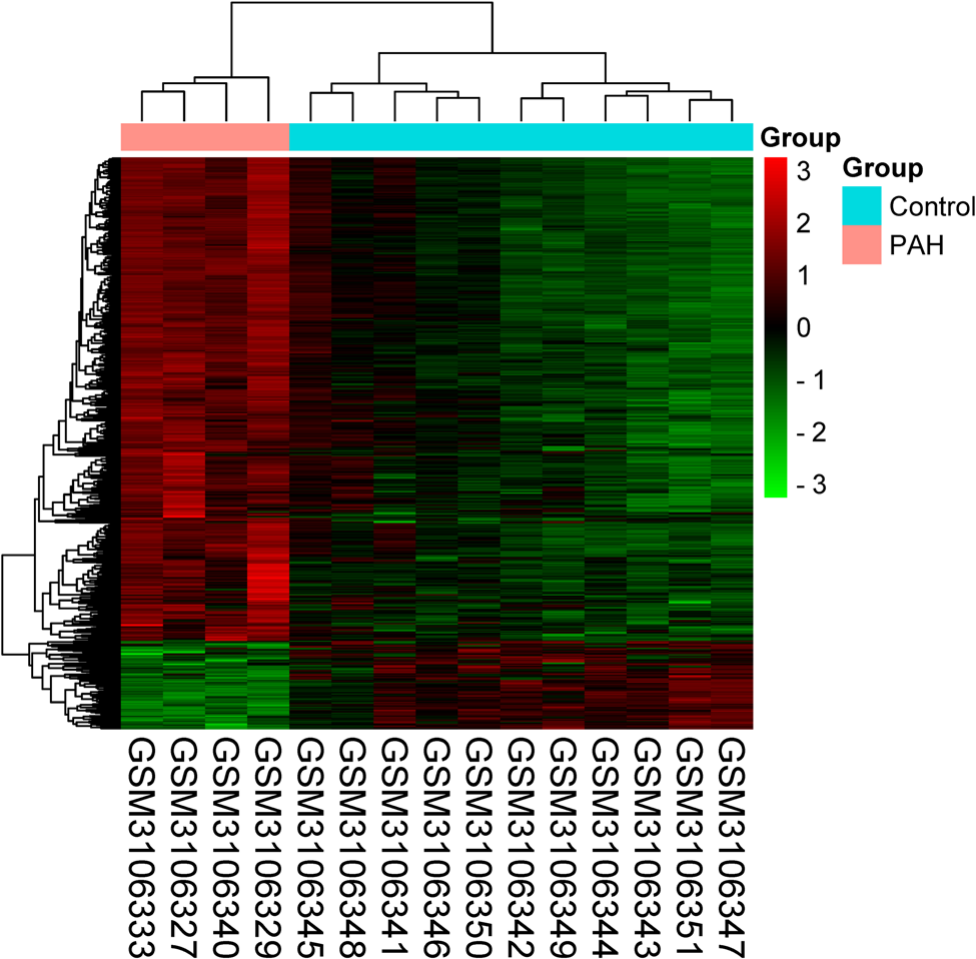
Clustered heatmap of DEGs in GSE113439 dataset. The green signifies downregulation, whereas the red represents the upregulation of DEGs.

**Figure 2:**
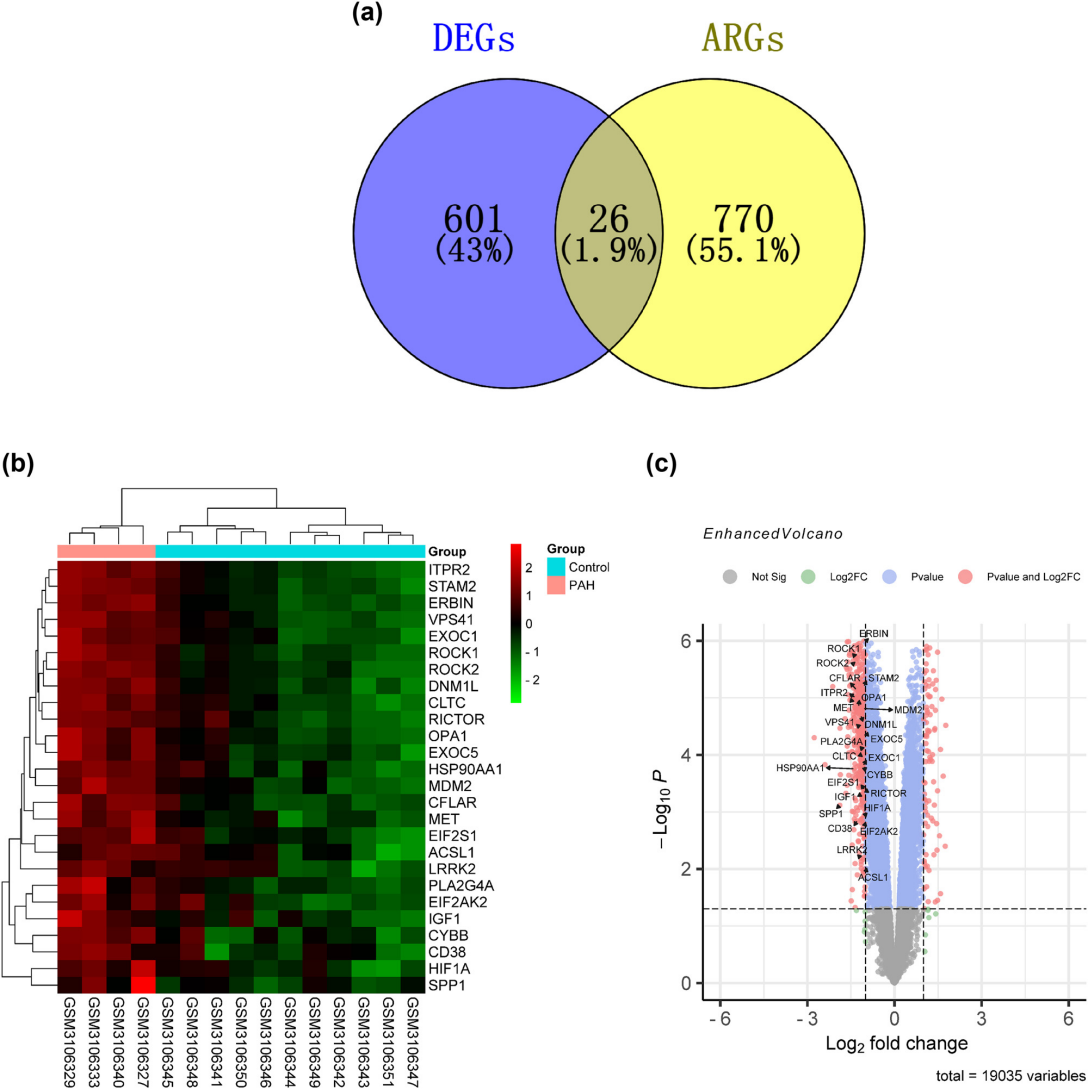
Identification of autophagy-related genes in patients with PAH and CHD: (a) Venn map indicating the intersection genes of ARGs and DEGs in GSE113439, (b) clustered heatmap of DEARGs in GSE113439, and (c) volcano plot of all genes in GSE113439, DEARGs marked in the diagram.

We identified 627 DEGs between control samples and PAH samples in the GSE113439 dataset, including 530 upregulated genes and 97 downregulated genes. The heatmap of 627 DEGs are presented in Figure 1. Next, we collected 796 ARGs from the Human Autophagy-dedicated Database. A total of 26 DEARGs were obtained by combining the results of 627 DEGs and 796 ARGs (Figure 2a). The heatmap and volcano plot of 26 DEARGs are presented in Figure 2b and c. Interestingly, all the DEARGs were upregulated.

### 3.2 Enrichment analysis of DEARGs

**Figure 3:**
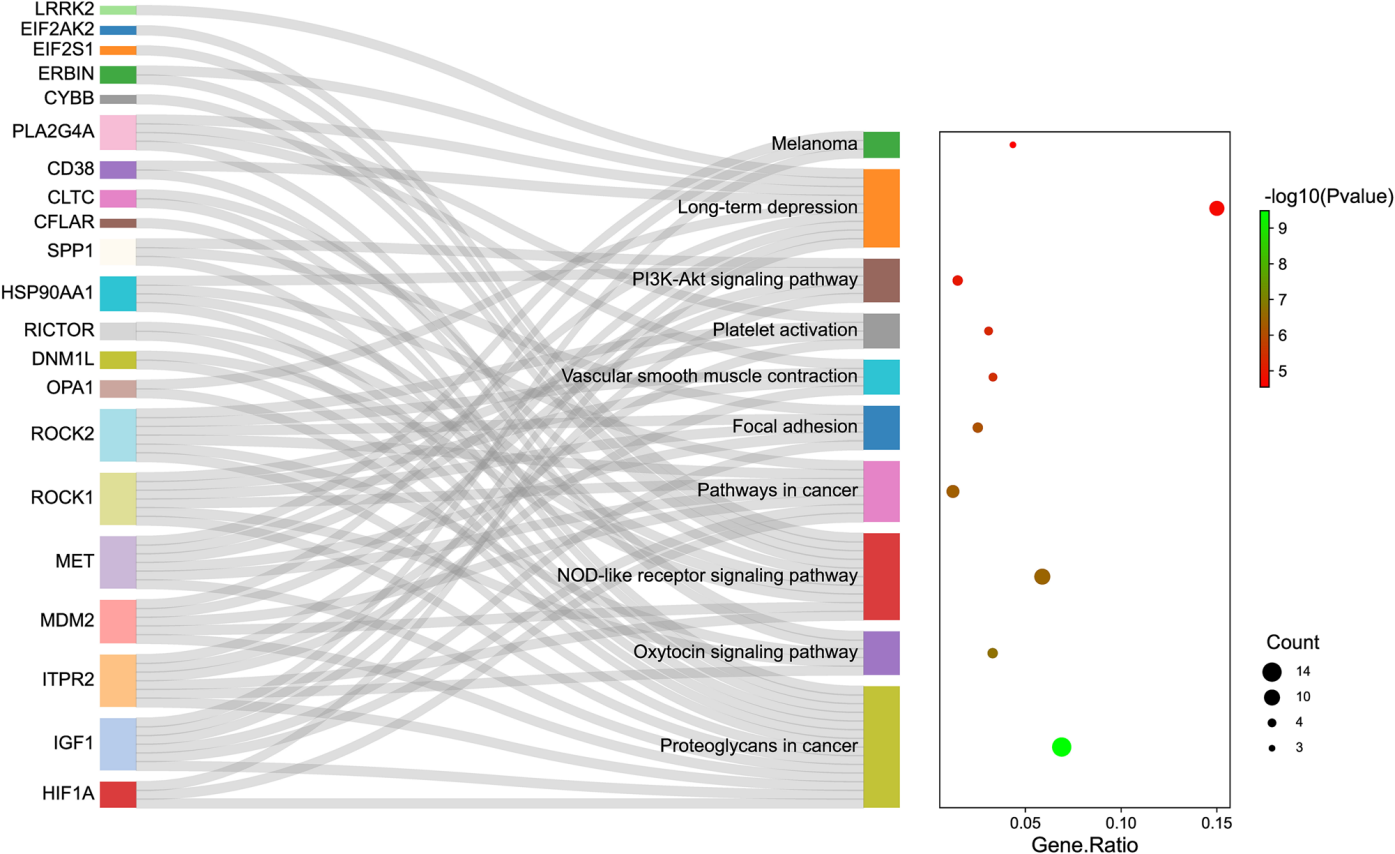
Sankey plot of KEGG enrichment analysis of DEARGs.

**Figure 4:**
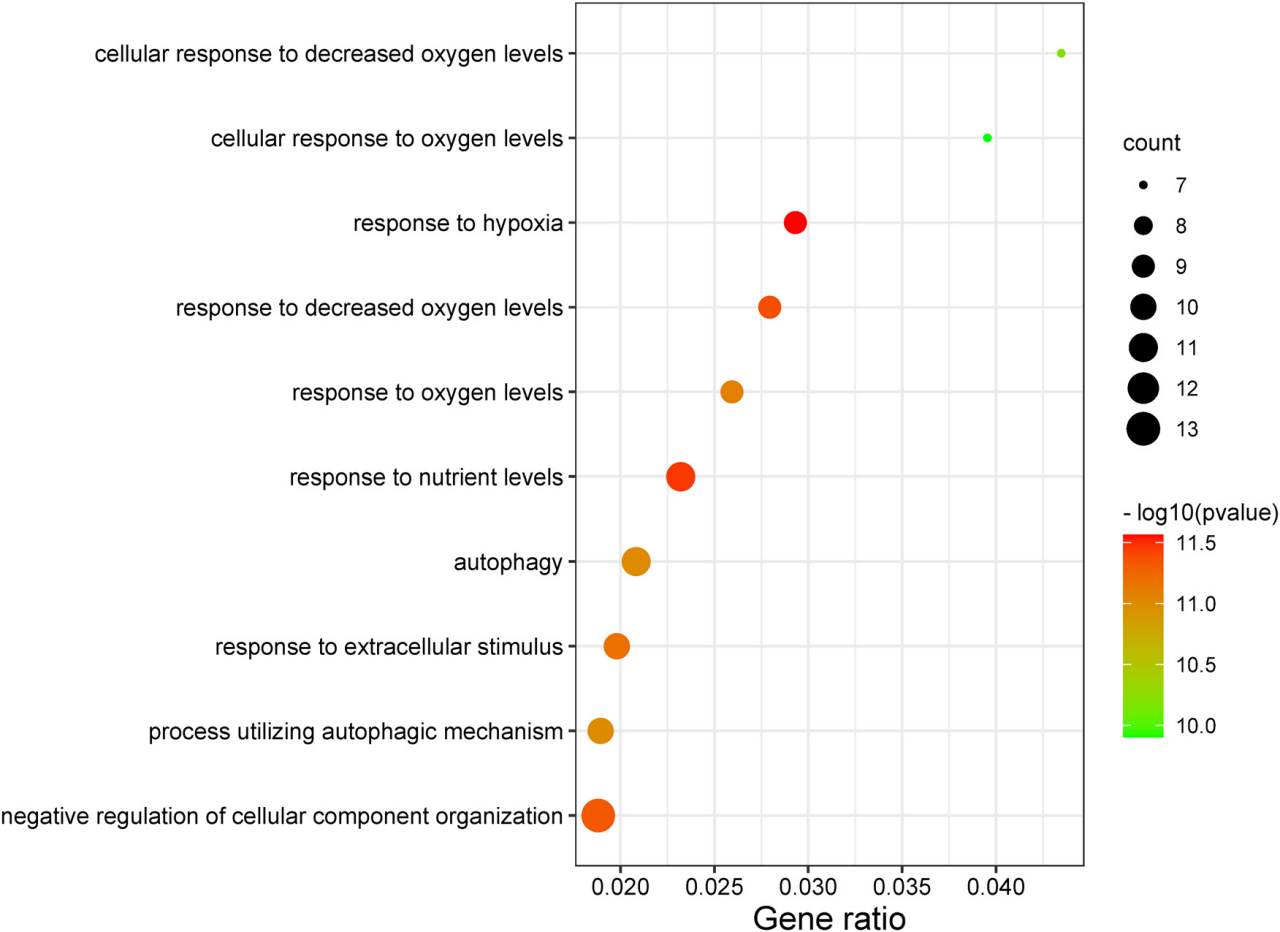
Bubble diagram of GO-BP enrichment analysis of DEARGs.

We performed functional enrichment analyses, including KEGG and GO-BP enrichment analyses to further investigate the biological function of DEARGs in PAH. Our results revealed that DEARGs were significantly enriched in proteoglycans in cancer, oxytocin signaling pathway, nucleotide-binding oligomerization domain (NOD)-like receptor signaling pathway, pathways in cancer, focal adhesion, vascular smooth muscle contraction, platelet activation, and PI3K-Akt signaling pathway. As presented in Figure 3 and Table 1, DEARGs such as IGF1, ITPR2, PLA2G4A, HIF1A, MET, OPA1, ERBIN, CD38, and LRRK2 were the ones involved in long-term depression. DEARGs such as CYBB, HSP90AA1, ITPR2, DNM1L, ERBIN, MDM2, EIF2S1, EIF2AK2, CLTC, and RICTOR were the ones associated with the NOD-like receptor signaling pathway. DEARGs such as HSP90AA1, IGF1, MDM2, MET, and SPP1 were the ones involved in the PI3K-Akt signaling pathway. In GO-BP analysis, the DEARGs were mainly associated with the processes of response to hypoxia, response to nutrient levels, response to decreased oxygen levels, negative regulation of cellular component organization, response to extracellular stimulus, response to oxygen levels, autophagy, a process utilizing autophagic mechanism, etc. (Figure 4 and Table 2).

**Table 1:** KEGG analysis of ARDEGs in PAH (top ten listed)

Description	Gene ratio	*P* value	Symbols	Count
Proteoglycans in cancer	0.0689655	3.26789 × 10^−10^	HIF1A/IGF1/ITPR2/MDM2/MET/ROCK1/ROCK2/OPA1/	14
			DNM1L/RICTOR/HSP90AA1/SPP1/CFLAR/CLTC	
Oxytocin signaling pathway	0.0328947	1.81365 × 10^−7^	CD38/ITPR2/PLA2G4A/ROCK1/ROCK2	5
NOD-like receptor signaling pathway	0.0588235	3.16322 × 10^−7^	CYBB/HSP90AA1/ITPR2/DNM1L/ERBIN/MDM2/EIF2S1/EIF2AK2/CLTC/RICTOR	10
Pathways in cancer	0.0121528	4.21189 × 10^−7^	HIF1A/HSP90AA1/IGF1/MDM2/MET/ROCK1/ROCK2	7
Focal adhesion	0.0251256	6.8965 × 10^−7^	IGF1/MET/ROCK1/SPP1/ROCK2	5
Vascular smooth muscle contraction	0.0330579	3.4048 × 10^−6^	ITPR2/PLA2G4A/ROCK1/ROCK2	4
Platelet activation	0.0307692	4.52862 × 10^−6^	ITPR2/PLA2G4A/ROCK1/ROCK2	4
PI3K-Akt signaling pathway	0.0146199	9.72001 × 10^−6^	HSP90AA1/IGF1/MDM2/MET/SPP1	5
Long-term depression	0.15	1.86851 × 10^−5^	IGF1/ITPR2/PLA2G4A/HIF1A/MET/OPA1/ERBIN/CD38/	9
			LRRK2	
Melanoma	0.0434783	2.84622 × 10^−5^	IGF1/MDM2/MET	3

**Table 2:** GO-BP analysis of ARDEGs in PAH (top ten listed)

Description	Gene ratio	*P* value	Symbols	Count
Response to hypoxia	0.029315961	2.73462 × 10^−12^	CD38/CYBB/HIF1A/ITPR2/MDM2/OPA1/CFLAR/ROCK2/	9
			DNM1L	
Response to nutrient levels	0.023206751	3.45527 × 10^−12^	CYBB/EIF2S1/ACSL1/MDM2/OPA1/EIF2AK2/SPP1/DNM1L/	11
			VPS41/LRRK2/CLTC	
Response to decreased oxygen levels	0.027950311	4.19225 × 10^−12^	CD38/CYBB/HIF1A/ITPR2/MDM2/OPA1/CFLAR/ROCK2/	9
			DNM1L	
Negative regulation of cellular component organization	0.018813314	4.63384 × 10^−12^	CD38/IGF1/MDM2/MET/OPA1/ROCK1/SPP1/CFLAR/ROCK2/	13
			DNM1L/LRRK2/CYBB/ITPR2	
Response to extracellular stimulus	0.01980198	6.45082 × 10^−12^	CYBB/EIF2S1/ACSL1/MDM2/OPA1/EIF2AK2/SPP1/DNM1L/	10
			VPS41/LRRK2	
Response to oxygen levels	0.025936599	8.17951 × 10^−12^	CD38/CYBB/HIF1A/ITPR2/MDM2/OPA1/CFLAR/ROCK2/	9
			DNM1L	
Autophagy	0.020833333	9.99713 × 10^−12^	CLTC/HIF1A/HSP90AA1/MET/ROCK1/DNM1L/STAM2/VPS41/EXOC1/LRRK2/PLA2G4A	11
Process utilizing autophagic mechanism	0.018939394	9.99713 × 10^−12^	CLTC/HIF1A/HSP90AA1/MET/ROCK1/DNM1L/STAM2/VPS41/EXOC1/LRRK2	10
Cellular response to decreased oxygen levels	0.043478261	6.42147 × 10^−11^	CYBB/HIF1A/MDM2/OPA1/CFLAR/ROCK2/DNM1L	7
Cellular response to oxygen levels	0.039548023	1.25051 × 10^−10^	CYBB/HIF1A/MDM2/OPA1/CFLAR/ROCK2/DNM1L	7

### 3.3 PPI network of analysis

**Figure 5:**
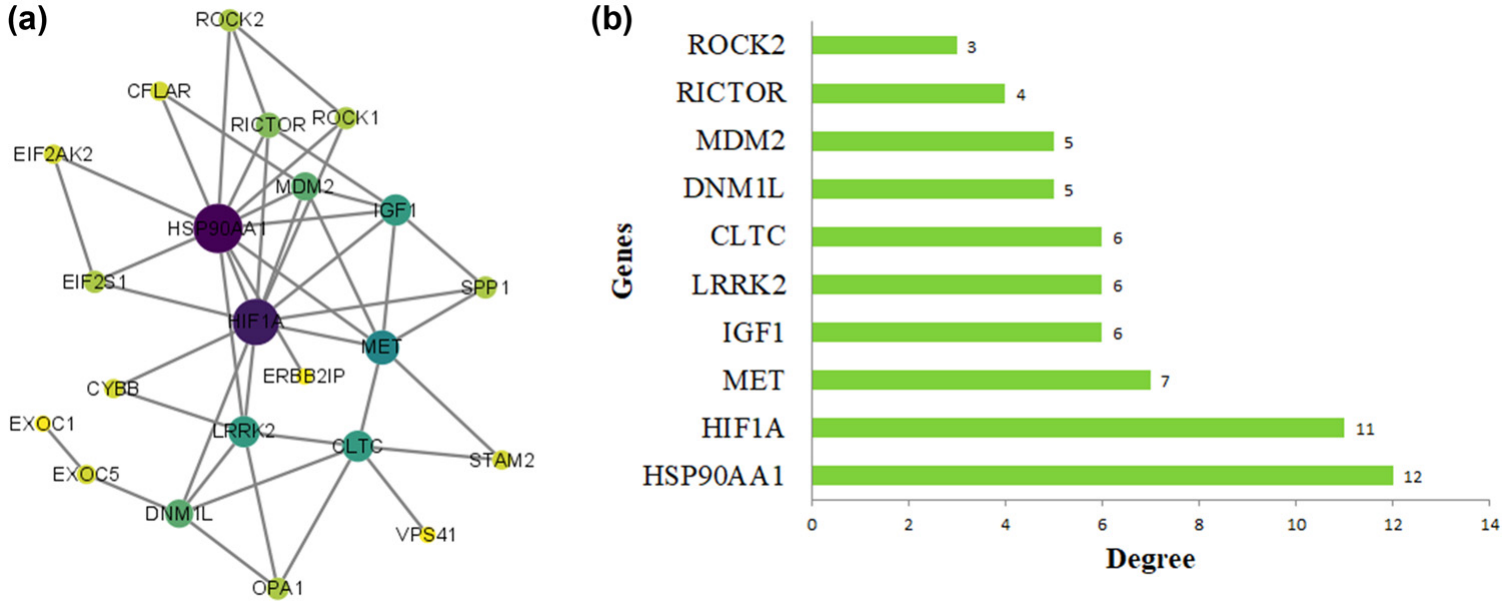
PPI network analysis of the DEARGs: (a) PPI network of DEARGs. The color and size of nodes indicate the value of a degree, the larger and deeper a node, the more important it is in the network. (b) Top ten genes of DEARGs by degree score ranking.

In this study, we established a PPI network of DEARGs based on the STRING database and Cytoscape software to investigate the interactions of these targets and screen important targets of the network. As shown in Figure 5a, a PPI network contains 22 nodes and 45 edges. These DEARGs were ranked based on their degree values, and the top ten genes with the highest degrees, namely HSP90AA1, HIF1A, MET, IGF1, LRRK2, CLTC, DNM1L, MDM2, RICTOR, and ROCK2 (Figure 5b).

### 3.4 Validation of hub genes expression

**Figure 6:**
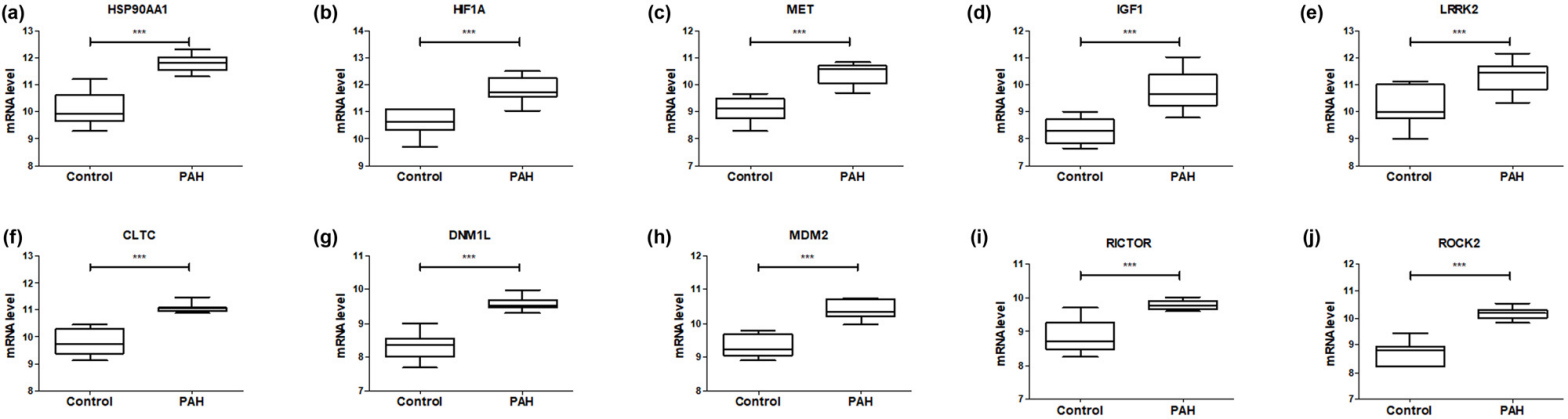
Verification expression levels of hub genes in GSE53408 dataset: (a) HSP90AA1, (b) HIF1A, (c) MET, (d) IGF1, (e) LRRK2, (f) CLTC, (g) DNM1L, (h) MDM2, (i) RICTOR, and (j) ROCK2. GSE53408 contains 11 healthy individuals and 12 severe pulmonary arterial hypertension patients. ***P < 0.001.

The expression levels of hub genes in the GSE53408 dataset were validated to further confirm the reliability of the GSE113439 dataset. As shown in Figure 6, the expression levels of HSP90AA1, HIF1A, MET, IGF1, LRRK2, CLTC, DNM1L, MDM2, RICTOR, and ROCK2 were significantly lower in control samples than in PAH samples.

### 3.5 ROC curve analysis

**Figure 7:**
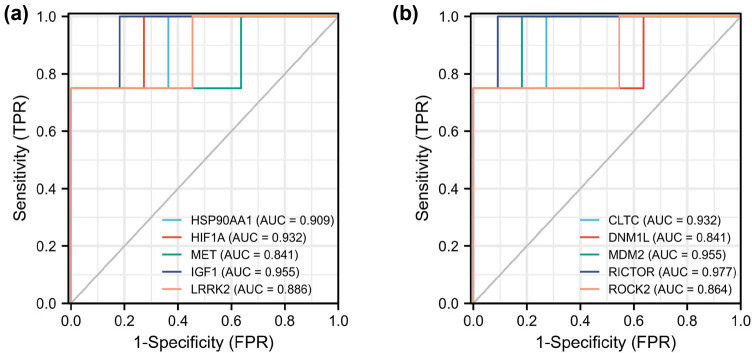
ROC analysis of ten hub genes: (a) the ROC curves of HSP90AA1, HIF1A, MET, IGF1, and LRRK2 in the diagnosis of PAH and (b) the ROC curves of CLTC, DNM1L, MDM2, RICTOR, and ROCK2 in the diagnosis of PAH.

To assess the sensitivity and specificity of the potential target for the diagnosis of PAH, the ROC analysis was performed. As a result, the AUC values of ten hub genes for PAH exceeded 0.8 in the GSE113439 dataset (Figure 7), which verifies the diagnostic value of hub genes (HSP90AA1, HIF1A, MET, IGF1, LRRK2, CLTC, DNM1L, MDM2, RICTOR, and ROCK2) in PAH and indicates these hub genes may play a vital role in PAH pathogenesis.

## 4 Discussion

PAH is a serious cardio-respiratory disease evoked by multiple risk factors [20]. Epidemiological research has revealed that the 5-year overall survival rate of PAH sufferers is less than 60% [21]. Therefore, accurate classification and diagnosis are the necessary means to improve the overall survival rate. Although studies on PAH have increased and relevant studies have made some progress, the specific pathological mechanism of PAH is still unknown and the clinical treatment effect is not ideal [22]. Autophagy has been reported to be a key regulator in human pulmonary pathologies and is considered a potential therapeutic target [23]. The interplay between reactive oxygen species and autophagy plays a complex and important role in the pathogenesis of pulmonary diseases, including PAH [24]. Previous reports have indicated that pulmonary artery smooth muscle cell autophagy is associated with pulmonary vascular remodeling and implicated in the pathological process of PAH [25,26]. However, the role of autophagy in the pathological mechanism of PAH needs to be further investigated.

With the development of a high-throughput gene assay, genomics research has been carried out to investigate the pathogenesis of PAH. In this study, we employed bioinformatics approaches to analyze the gene expression profiles of the GSE113439 dataset and identified 26 DEARGs associated with the pathological mechanism of PAH. Besides, the top ten hub genes were further identified and verified by using the GSE53408 dataset.

We also performed the KEGG and GO-BP enrichment analyses to reveal the potential biological function of DEARGs in PAH. The findings of KEGG analysis indicated that the DEARGs were mainly enriched in the PI3K-AKT and NOD-like receptor signaling pathways, which were to the previous results that PI3K-AKT and NOD-like receptor were key signaling pathways in PAH [27,28]. Additionally, GO-BP analysis revealed that the DEARGs were markedly enriched in the autophagy and response to oxygen levels. Previous studies have demonstrated that autophagy is involved in the progression of PAH. For example, liraglutide has been shown to ameliorate PAH through the regulation of autophagy pathways [29]. NSD2 silencing could ameliorate PAH via inhibiting pulmonary artery autophagy [16]. Besides, autophagy activation helps with BMPR2 degradation and it plays a crucial role in the development of PAH [30].

In this study, ten hub genes (HSP90AA1, HIF1A, MET, IGF1, LRRK2, CLTC, DNM1L, MDM2, RICTOR, and ROCK2) associated with PAH were identified via PPI network analysis. We further confirmed the expression level of hub genes by using the GSE53408 dataset. These results were consistent with the bioinformatics analysis findings from the GSE113439 dataset. In addition, these ten DEARGs showed a high diagnosis value in PAH. To date, few studies have reported that these genes are involved in the pathogenesis of pulmonary diseases via the regulation of autophagy. However, several studies have reported them to be related to pulmonary diseases or autophagy. For example, a recent study demonstrated that heat shock protein 90 α family class A member 1 (HSP90AA1) induces autophagy via inactivation of the AKT-MTOR pathway [31]. HSP90AA1 is a novel therapeutic target in PAH [32]. A previous study reported degradation of hypoxia-inducible factor 1 subunit alpha (HIF1A) in the lysosome via chaperone-medicated autophagy [33]. HIF1A was reported to play a vital role in hypoxia-induced pulmonary hypertension [34]. A higher level of HIF1A in human pulmonary artery smooth muscle cells could increase susceptibility to developing PAH [35]. Mesenchymal-epithelial transition (MET) is important for autophagy regulation through the mTOR pathway [36]. MET has not been reported in PAH. However, the MET signaling pathway has been demonstrated as a potential pathway in non-small-cell lung cancer [37]. An inhibitory effect of insulin-like growth factor 1 (IGF1) on autophagy has been found in rat cardiomyocytes [38]. A previous report indicated that IGF1 prevents cell apoptosis in pulmonary artery smooth muscle cells during PAH [39]. IGF1 was also implicated in the pathogenesis of PAH in neonatal mice [40]. Leucine-rich repeat kinase 2 (LRRK2) has been involved in autophagy [41]. It has not been reported in PAH, but a previous report revealed that it might be a key gene that helped tumorigenesis in non-small-cell lung cancer [42,43]. Alias for the DNM1L gene is dynamin-related protein 1 (DRP1). DRP1-dependent mitochondrial fission promotes pulmonary vascular remodeling via activating autophagy, indicating that DNM1L might be a therapeutic target for the treatment of PAH [44]. Murine double minute (MDM2) contributes to mitophagy of damaged mitochondria [45]. It has been reported that MDM2 and AMPK crosstalk is involved in the pathogenesis of PAH, and a combined intervention of MDM2 and AMPK might be a novel therapy in PAH treatment [46]. Rapamycin-insensitive companion of mTOR (RICTOR) amplification promotes non-small cell lung cancer proliferation via generation of mTORC2 [47]. Pharmacologic inhibition of Rho-associated protein kinase 2 (ROCK2) could induce autophagy [48]. ROCK2 regulates the cell cycle progression in hypoxia-induced PAH, suggesting it might be a novel target for improving PAH treatment [49]. To date, there is no relevant research that reported clathrin 1 gene (CLTC) is associated with lung diseases. Although potential DEARGs associated with PAH were identified based on bioinformatics, the experimental studies should be performed both *in vitro* and *in vivo* to further verify our findings.

## 5 Conclusion

Overall, our findings revealed HSP90AA1, HIF1A, MET, IGF1, LRRK2, CLTC, DNM1L, MDM2, RICTOR, and ROCK2 as potential diagnostic biomarkers and therapeutic targets for PAH. These findings broaden our understanding of the pathogenesis of PAH.
